# Supplementary figures and images for: Amplicon Sequencing Analysis of Submerged Plant Microbiome Diversity and Screening for ACC Deaminase Production by Microbes
Source: Int J Mol Sci. 2024 Dec 12;25(24):13330. doi: 10.3390/ijms252413330 (PMC11727893; doi:10.3390/ijms252413330)

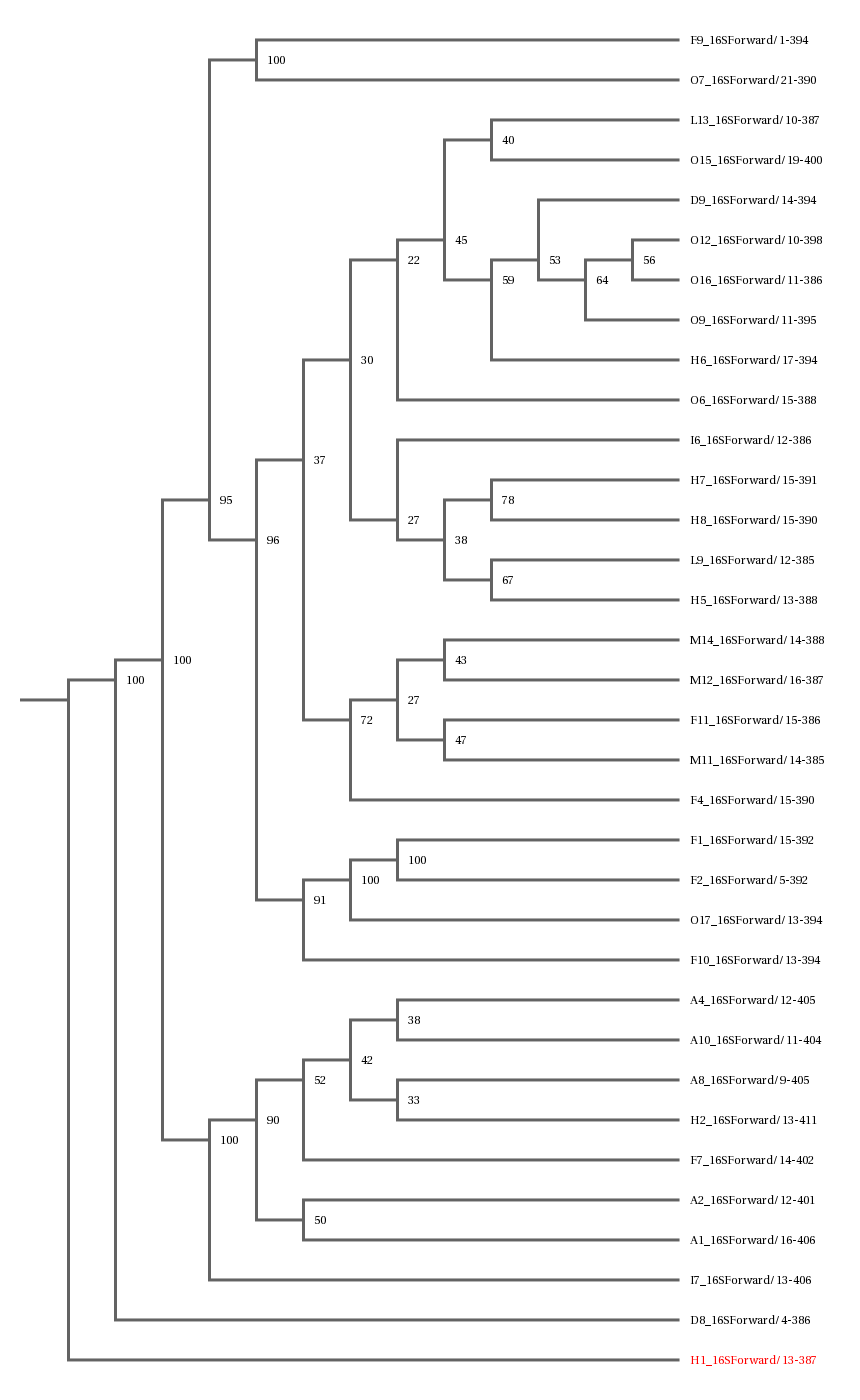

Supplement: Supplementary file 1 [file ijms-25-13330-s001.zip › Figure S1 Phyllogenetic tree.png]
